# Is Membrane Filtration Applicable for the Recovery of Biologically Active Substances from Spent Lavender?

**DOI:** 10.3390/membranes15010021

**Published:** 2025-01-11

**Authors:** Yoana Stoyanova, Nevena Lazarova-Zdravkova, Dimitar Peshev

**Affiliations:** 1 Department of Biotechnology, University of Chemical Technology and Metallurgy, 1576 Sofia, Bulgaria; stoyanova@uctm.edu (Y.S.); nevena@uctm.edu (N.L.-Z.); 2 Department of Chemical Engineering, University of Chemical Technology and Metallurgy, 1576 Sofia, Bulgaria

**Keywords:** spent lavender, membrane filtration, HPLC, antioxidant activity, antibacterial activity

## Abstract

This study explored the batch membrane filtration of 40% ethanol extracts from spent lavender, containing valuable compounds like rosmarinic acid, caffeic acid, and luteolin, using a polyamide-urea thin film composite X201 membrane. Conducted at room temperature and 20 bar transmembrane pressure, the process demonstrated high efficiency, with rejection rates exceeding 98% for global antioxidant activity and 93–100% for absolute concentrations of the target components. During concentration, the permeate flux declined from 2.43 to 1.24 L·m^−2^·h^−1^ as the permeate-to-retentate-volume ratio increased from 0 to 1. The process resistance, driven by osmotic pressure and concentration polarization, followed a power–law relationship with a power value of 1.20, consistent with prior nanofiltration studies of rosmarinic acid solutions. Notably, no membrane fouling occurred, confirming the method’s scalability without compromising biological activity. The antioxidant activity, assessed via the DPPH method, revealed that the retentate exhibited double the activity of the feed. Antibacterial assays using broth microdilution showed that the retentate inhibited *Escherichia coli* by 73–96% and *Bacillus subtilis* by 97–98%, making it the most active fraction. These findings validate the effectiveness of the X201 membrane for concentrating natural antioxidants and antibacterial agents from lavender extract under sustainable operating conditions.

## 1. Introduction

*Lavandula angustifolia* is considered a pharmacopoeia raw material with a valuable medicinal effect [1,2]. Lavender is traditionally believed to exhibit antioxidant [3], anti-inflammatory [4,5,6,7], sedative [8], antidepressant [9], spasmolytic, anticholinesterase [10], antifungal [7], and antibacterial [1] properties.

Growing and processing lavender is an emblematic industry in Bulgaria, which has a long-standing tradition in the production of essential oils. Besides being a country known for the production of rose oil, in recent years, Bulgaria has become the world leader in the production of lavender oil.

Steam distillation is the primary method of ensuring the high and consistent quality of lavender essential oil. During the distillation of lavender essential oil, three waste fractions are generated as the following: aqueous condensate (hydrolate, hydrosol), wastewater (so-called residue), and spent plant mass [11].

The spent lavender is often overlooked despite its potential to contain a rich array of valuable bioactive compounds [11,12,13,14]. It contains biologically active phytochemicals such as flavonoids, phenolic acids, and tannins, which have been studied for their antioxidant, antimicrobial, and anti-inflammatory properties. Key components found in spent lavender include rosmarinic acid, which is noted for its potent antioxidant and anti-inflammatory properties and plays a crucial role in protecting cells from oxidative damage [11,12,13,15,16,17,18]. Caffeic acid, known for its ability to scavenge free radicals, contributes to the overall antioxidant capacity of lavender waste extracts [12,15,16,17,18]. Additionally, luteolin, a flavonoid, exhibits both antioxidant and antimicrobial activities, making it a valuable functional ingredient in food supplements and medicines [12,13,14,18]. Moreover, waste lavender materials contain other flavonoids, such as apigenin [12,13,14] and hesperidin [19], possessing antioxidant properties and health-promoting potential.

Research indicates that waste lavender extracts effectively inhibit the growth of various pathogenic bacteria [16,20] and fungi [16], highlighting their potential use as natural preservatives in food and cosmetic products. For instance, studies have shown that extracts can inhibit the growth of *Escherichia coli* [16,21] and *Bacillus subtilis* [16].

Every year, in the territory of Bulgaria, the production of lavender oil generates huge amounts of waste materials, which in areas with intensive essential oil industry become an environmental problem. Uncontrolled disposal of these waste fractions with potential biological activity leads to contamination of the soil as well as the surface and groundwater. In addition, valuable substances with biological activity are lost.

Separation processes at the molecular level using reverse osmosis membranes offer several advantages over traditional methods. Current research trends in the development of membrane filtration technology emphasize its application in wastewater treatment and clean water production [22]. The efficiency of membrane separation techniques, particularly membrane filtration, is being investigated as a sustainable method for processing biologically active liquid extracts. The selectivity of nanofiltration and reverse osmosis membranes enables efficient molecular separations at low or ambient temperatures, preserving heat-sensitive compounds such as polyphenols and flavonoids, which are susceptible to degradation under thermal stress and can lose their therapeutic properties entirely. In contrast to commonly used thermal processes, such as distillation and evaporation, membrane filtration provides significant energy savings by allowing separation without a phase transition. Furthermore, the permeate obtained from nanofiltration or reverse osmosis of the natural extracts can be reused as a solvent during the solvent extraction, thereby facilitating a closed cycle in essential oil production.

In recent years, extensive research has been conducted on the utilization of by-products in the agricultural and food industries [23,24,25,26,27,28,29]. However, there is a lack of information regarding the application of membrane filtration for the recovery of biologically active substances contained in the waste plant material from the production of lavender essential oil.

This study aims to demonstrate that the selected reverse osmosis PA-Urea-TFC X201 membrane exhibits the necessary rejection capability, permeate flux, and resistance to instantaneous fouling during the filtration of hydroalcoholic antioxidant extracts from spent lavender. Complete rejection of key biologically active constituents, reasonable flux, and resistance to fouling would justify further research on the design of membrane concentration processes, ensuring lower specific energy consumption and biological activity loss in comparison with traditional thermal separation techniques.

## 2. Materials and Methods

### 2.1. Materials

The spent lavender was obtained from Galen N Ltd. (Zelenikovo, Bulgaria). *Lavandula angustifolia* was grown in the region of Chirpan, Bulgaria. The spent lavender was collected immediately after the essential oil extraction via steam distillation in the industrial facility of Galen N Ltd. (Zelenikovo, Bulgaria). The plant material consisted of stems, flowers, and leaves. The material was dried at ambient temperature in a dry, well-ventilated place, avoiding direct sunlight. The dried waste material was milled and kept in airtight containers in a dark place until use. The particle size distribution of the powder material was measured and reported in a previous publication [30]. The mean diameter of 50% of the particles (d_50.3_) was less than 563 ± 106 μM, whereas the mean Sauter diameter of the spent lavender was 218 ± 26 μM [30].

The absolute ethanol (99%) used for antioxidant activity determination was purchased from Valerus Ltd. (Sofia, Bulgaria) and 2,2-diphenyl-1-picryl-hydrazyl (DPPH•) from Sigma–Aldrich (Darmstadt, Germany). The solutions and standards used for HPLC analysis were supplied as the following: methyl alcohol, anhydrous, and acetonitrile (ChromaAR HPLC Super Gradient, from Macron Fine Chemicals (Phillipsburg, NJ, USA)), deionized water (ChromaAR HPLC, from Macron Fine Chemicals (Phillipsburg, NJ, USA)), rosmarinic acid (>97%, from Sigma Aldrich (Darmstadt, Germany)), caffeic acid (>98%, from Sigma Aldrich (Darmstadt, Germany)), and luteolin (97%, from Alfa Aesar (Ward Hill, MA, USA)). All reagents used were of analytical grade. The reverse osmosis polyamide urea thin film composite (PA-Urea-TFC) membrane X201 (99.5% retention of NaCl) used during the runs was provided by Trisep (Goleta, CA, USA). Microfiltration cellulose acetate membrane filter disks with a pore size of 0.45 µm and a diameter of 90 mm (Chemplus Scientific Ltd., Danyang, China) were supplied from Biotechlab (Sofia, Bulgaria). The following four media were used for antibacterial testing: Luria Agar Base, Luria Broth Base (LB), Nutrient Agar, and Nutrient Broth (NB). They were sourced from HiMedia (Mumbai, India). The test cultures, *Bacillus subtilis NBIMCC 3562* and *Escherichia coli NBIMCC K12 407*, were provided by the National Bank for Industrial Microorganisms and Cell Cultures (NBIMCC) in Sofia, Bulgaria.

### 2.2. Methods

#### 2.2.1. Solid–Liquid Extraction

The antioxidant liquid extracts from spent lavender in this work were obtained following a previously reported methodology [30]. The extraction of the powdered spent lavender was accomplished at optimal conditions: solid-to-liquid ratio of 1 g dry plant material to 10 mL of 40 vol% ethanol in water; extraction temperature of 30 ± 1 °C; 120 min extraction time; and intensive mixing ensuring no external mass transfer resistance to the solvent extraction.

#### 2.2.2. Membrane Filtration

**Figure 1:**
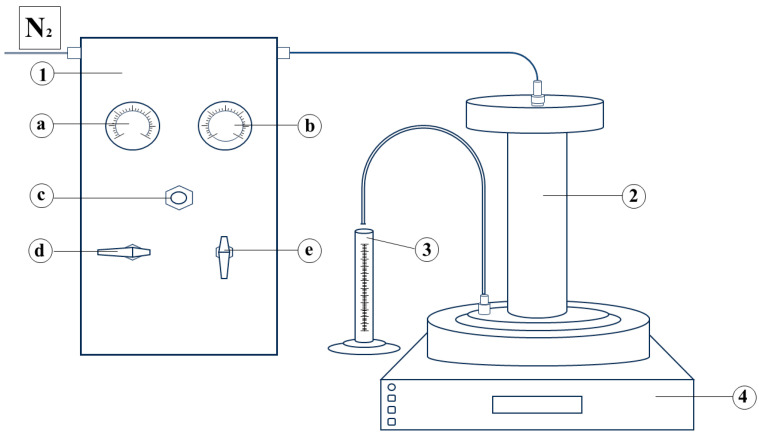
Laboratory set-up for batch membrane filtration: 1—pressure control unit; a—supply pressure gauge; b—membrane filtration cell pressure gauge; c—pressure regulation valve; d—isolation valve; e—vent valve; 2—membrane filtration cell; 3—permeate collection vessel; 4—electromagnetic stirrer.

A lab-scale membrane filtration experiment was conducted using a dead-end filtration cell (METcell, Evonik Membrane Extraction Technology, London, UK). This filtration cell is designed to test flat sheet polymeric membrane samples with a circular surface area of 54 cm^2^. The system operates at pressures of up to 69 bar, applied using compressed nitrogen (Figure 1), with a maximum feed volume capacity of 250 mL.

In order to minimize concentration polarization, the stirrer speed was kept constant at 350 rpm. The experiment was carried out at ambient temperature (20 ± 2 °C) and a pressure of 20 bar, maintained by high-purity nitrogen (99.996%) supplied from a cylinder. Before membrane filtration, the feed solution was microfiltered in the MET cell membrane filtration system to remove microscopic solids and prevent mechanical clogging of the membrane during reverse osmosis filtration. To avoid the “memory” effect on the membrane properties, each experiment was carried out with a new membrane sample. According to the instructions of the dead-end filtration cell manufacturer [31], the reverse osmosis membrane required pretreatment before use. Before each filtration, the membrane was conditioned by initially permeating 40% ethanol at 20 bar until a steady-state permeate flux was observed and at least 150 mL of permeate was collected. This step aimed to avoid the compression effect in the later stages of the experiments and to remove the conditioning agent used to preserve the membrane structure. In all membrane batch concentration experiments, 100 mL of the 40% ethanol extract of spent lavender was fed into the MET cell. During the batch concentration, the permeate was continuously collected in a cylinder, and the time for the accumulation of 50 mL of the permeate was measured. Hence, the volume of retentate remaining in the cell was also 50 mL. At the end of the process, samples of permeate and retentate were taken for determination of the rosmarinic acid, caffeic acid, and luteolin content, as well as for analysis of the biological activity. To prove that the reverse osmosis membrane did not alter the concentration of the hydroalcoholic solvent, additional batch membrane filtration experiment with 40 vol% ethanol as a feed was conducted under the same operating conditions as with the real extracts. The content of ethanol in the resulting permeate and retentate fractions was determined via gas chromatography (GC).

#### 2.2.3. HPLC and GC Analyses

The key bioactive compound (KBAC) content in the 40% ethanolic extract of spent lavender was determined using high-performance liquid chromatography (HPLC). A significant number of works related to the processing or application of natural extracts containing KBACs, such as rosmarinic acid, caffeic acid, luteolin, carnosol, etc., relied on HPLC analysis for their quantification [30,32,33,34,35,36,37,38]. The method used in the present study was adapted from the literature [30,37,38] and was also previously implemented by the research team [30].

For the KBAC content determination, a ternary Hewlett Packard (HP) Series II 1090 liquid chromatography system equipped with a UV-Vis detector (DAD) was used. The separation was carried out on an Agilent C18 column (15 mm × 4.6 mm, 5 μM particle size). The assay conditions were 30 °C, 0.7 mL/min mobile phase flow rate, and 5 μL injection volume. A mixture of solvent A (75 mL acetonitrile + 420 mL water + 4.25 mL acetic acid) and solvent B (methanol) was used as a mobile phase. To achieve a sufficient separation, a method with a 105 min duration and a variable solvent gradient was developed according to the following conditions: (1) 0–90 min, B: 0–100%; (2) 90–103 min, B: 100–100%; (3) 103–104 min, B:100–0%; (4) 104–105 min, B: 0–0%. Two detection wavelengths were preset as the following: 330 nm for rosmarinic and caffeic acids and 360 nm for luteolin. The peaks of the target compounds in the chromatograms of the obtained extracts were identified by comparing the retention times with those of their standards.

The HPLC method was calibrated with respect to KBACs by analyzing series of standard solutions using at least five levels of concentration, which covered the concentration range of 0–2 g/L for all compounds. The coefficients of linear correlation for all calibration lines were above 0.996.

The determination of ethanol content in the solvent was performed using a Shimadzu GC 2010 gas chromatograph with an AOC-20i autosampler and a FID detector under the following conditions: carrier gas, He; injector, temperature 240 °C; injected volume, 1 μL; split ratio, 1:50; makeup flow, 30 mL/min; H_2_ flow rate, 40 mL/min; air flow rate, 400 mL/min; purge flow rate, 3 mL/min; detector, temperature 250 °C; temperature gradient, 60 °C to 100 °C at a rate of 1°C/min and 100 °C to 220 °C at a rate of 20 °C/min.

#### 2.2.4. Antioxidant Capacity Determination

Various test methods have been used to evaluate the antioxidant activity of natural products [39]. Among all of them, the DPPH• radical scavenging assay is considered simple and reliable because it is stable, does not dimerize, reacts slowly enough with the entire sample over time, and is inexpensive. The DPPH method can be used in aqueous and nonpolar organic solvents. The results from the analysis are highly reproducible and comparable to other methods. Antioxidant efficiency is measured under normal conditions, thus eliminating the risk of the thermal decomposition of molecules [40]. DPPH• shows a strong absorption band in ethanol solution with a maximum at 517 nm, while the reduced form of DPPH-H does not absorb significantly at this wavelength, allowing a quantitative colorimetric determination [37,38,39,40,41].

The assay was developed as the following: an ethanolic solution of DPPH was prepared, and its concentration was adjusted to approximately 0.1 mM, so that the absorbance was about 0.88 AU at 517 nm. The DPPH solution in absolute ethanol was freshly prepared for each experiment. Single-use polystyrene cuvettes (3 mL capacity, 10 mm path length) and a T70 UV-Vis Spectrophotometer (PG Instruments Ltd., Lutterworth, UK) were used for measuring the absorbance.

To ensure a moderate radical scavenging reaction rate and equilibrium in the reaction mixture and therefore accurate analysis, appropriate dilutions of the studied extract from the spent lavender and its membrane-filtrated fractions were prepared. The concentration of the spent lavender extract and its reverse osmosis fractions used in the antioxidant capacity assay were the following: feed (extract)—1.54, 1.24, 0.896, 0.633, and 0.424 mL/L; Permeate—32.3, 16.1, 10.8, and 5.38 mL/L; Retentate—1.24, 0.896, 0.633, 0.424, 0.319, and 0.256 mL/L.

To determine the antioxidant activity of the extract from the spent lavender and its fractions, 0.05 mL of the tested sample, with a concentration as reported above, was added to 1.5 mL of DPPH• solution. The control sample contained 1.5 mL of DPPH• solution and 0.05 mL of absolute ethanol. After mixing the antioxidant sample and the DPPH• solution, the absorbance of the control (A_C_) and the test (A_S_) samples was measured at 517 nm every 15 min for 1 h in order to examine the kinetics of the reaction. Each measurement was performed in two replicates. The free radical scavenging activity of each sample was calculated as the deviation of the light absorbance for analyzed samples with respect to the control sample according to the following Equation (1):

(1) $$\Delta Abs=A_{c}-A_{s}$$

The deviation of the absorbance per milliliter of the undiluted extract from the spent lavender and its reverse osmosis fractions was calculated as the following (AU/mL extract):

(2) ∆AbsmL= ∆Abs × times dilution0.05

According to Equation (2), ΔAbs/mL is independent of the degree of sample dilution and the absorbance of the blank sample during the analysis, which enables its application for determining the absolute antioxidant capacities of the samples. The results are presented as the mean arithmetic values of two replicates for ΔAbs/mL. The deviation of the experimental points from the mean arithmetic was less than 7.8% in all cases.

#### 2.2.5. Antibacterial Activity

The broth microdilution method was used to measure the activity of the spent lavender extract and its fractions against the model strains of Gram-positive bacteria *Bacillus subtilis NBIMCC 3562* and the facultative anaerobic Gram-negative *Escherichia coli K12 NBIMCC 407*. A Microplate Reader PKL PPC 142 (Pokler, (Pontecagnano Faiano, Italy)) with a 96-well plate was used for the purpose of this study. The liquid nutrient media, Nutrient Broth (NB) for *B. subtilis 3562* and Luria Broth (LB) for *E. coli K12 407*, were prepared. Both culture media were sterilized in an autoclave at a temperature of 121 °C and a pressure of 1 atm, in order to destroy all vegetative life forms. Precultures of *B. subtilis 3562* and *E. coli K12 407* were prepared as the following: a single colony was selected from the Petri dish with a grown strain of *B. subtilis 3562* or *E. coli K14 407* and was carefully sieved into a 300 mL Erlenmeyer flask (filled with 50 mL of liquid medium—NB or LB). The culture, taken carefully with the inoculation needle, was smeared at the liquid–air interface. Then, the cultures were incubated in a Shaker ES-20/60 at 30 °C for *B. subtilis 3562* and 37 °C for *E. coli K12 407* for 18 h. Using a densitometer DEN-1B (Biosan, Riga, Latvia), the bacterial inoculum was diluted to 0.5 McFarland, which corresponds to approximately 1.5 × 10^8^ CFU/mL. For conducting the experiment, two types of liquid nutrient media were prepared. The first one was prepared with distilled water and the second one with aqueous solutions of the extract from the spent lavender or its reverse osmosis fractions with concentrations of 670, 550, and 440 mL/L. The control samples were prepared in standard NB and LB media, respectively. The concentrations of the extract from the spent lavender and its reverse osmosis fractions analyzed for their antibacterial activity are presented in Section 3.4. Bacterial growth was measured at a wavelength of 630 nm after 24 h of incubation. All measurements were performed in five replicates and the mean arithmetic values were used to calculate the percent reduction in live cells of the two tested strains in the presence of the spent lavender extract and its fractions.

## 3. Formularization, Results, and Discussion

### 3.1. Membrane Selectivity Against Total Antioxidant Activity and Key Biologically Active Constituents

**Figure 2:**
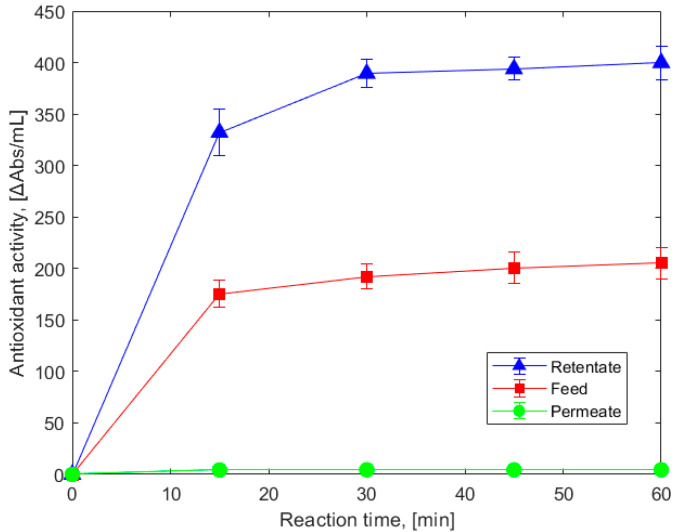
Kinetics of the radical scavenging reaction of spent lavender extract (Feed) and its fractions obtained by filtration with the X201 reverse osmosis membrane.

The kinetics of the DPPH• reaction with the antioxidants from the spent lavender extract and its reverse osmosis fractions were investigated with the aim to select a suitable reference time of reaction for the evaluation of the absolute radical scavenging capacity of the investigated samples. As shown in Figure 2, all three types of samples reacted rapidly with the DPPH• radical within 30 min, after which the reaction reached a plateau. To eliminate the impact of time and conduct an accurate comparative study of the antioxidant potential of the products, all samples were allowed to react for a standardized duration of 60 min in all subsequent measurements.

Figure 2 also displays the ΔAbs/mL values of the spent lavender extract and its fractions, as determined using Equations (1) and (2). The effectiveness of the membrane concentration of the three solutions was quantitatively assessed using the ΔAbs/mL values obtained at the 60th minute of the DPPH• quenching reaction for their test samples. As illustrated in Figure 2, membrane permeation of half the volume of the spent lavender extract at room temperature resulted in a retentate with antioxidant activity approximately twice that of the feed solution and permeate with negligible activity. This result indicates almost complete retention of the antioxidant constituents in the solutions, and at the same time, their complete recovery in the retentate fraction.

The ability of the reverse osmosis membrane to selectively retain antioxidant components in the spent lavender liquid extract was quantitatively characterized by the membrane rejection coefficients. These coefficients were determined according to two different definitions [30,42], firstly using the deviation in absorbance per milliliter (AU/mL) of the undiluted extract or its fractions (Equations (3) and (4)), and secondly using the absolute concentrations of the individual key components as determined through HPLC analysis (Equations (6)–(8)):

(3) $$R_{1}=\left(1-\frac{\frac{{∆Abs}_{P}}{{mL}_{P}}}{\frac{{∆Abs}_{F}}{{mL}_{F}}}\right)100,\;\%$$

(4) $$R_{2}=\left(1-\frac{\mathrm{lg}\left(\frac{{∆Abs}_{R}/{mL}_{R}}{{∆Abs}_{F}/{mL}_{F}}\right)}{lg\left(V_{F}/V_{R}\right)}\right)\;100,\;\%$$

(5) $$Err=\frac{V_{F\;}{∆Abs}_{F}/{mL}_{F}-[V_{P\;}{∆Abs}_{P}/{mL}_{P}+V_{R\;}{∆Abs}_{R}/{mL}_{R}]}{V_{F\;}{∆Abs}_{F}/{mL}_{F}}\;100,\;\%$$

(6) $$R_{3}=\left(1-\frac{C_{P}}{C_{F}}\right)100,\;\%$$

(7) $$R_{4}=\left(1-\frac{C_{PE}}{C_{R}}\right)\;100,\;\%\;$$

(8) $$R_{5}=\left(1-\frac{\mathrm{lg}\left(C_{R}/C_{F}\right)}{lg\left(V_{F}/V_{R}\right)}\right)100,\;\%$$

(9) $$Err=\frac{V_{F\;}C_{F\;}-[V_{P}C_{P}+V_{R}C_{R}]}{V_{F}C_{F}}\;100,\;\%$$

where ΔAbs_F_/mL_F_, ΔAbs_P_/mL_P_, and ΔAbs_R_/mL_R_ denote the deviation in absorbance per milliliter (AU/mL) for the undiluted spent lavender extract (feed), permeate, and retentate solutions. The variables *V_F_*, *V_P_*, and *V_R_* represent the volumes of these fractions, while *C_F_*, *C_P_*, and *C_R_* indicate the concentrations of target compounds in the feed, permeate, and retentate, respectively. *C_PE_* is the instantaneous concentration of the target compounds in the permeate just before the end of the batch membrane filtration.

Based on Equations (3) and (4), the membrane rejection coefficients, evaluated in terms of global antioxidant activity, were determined as *R*_1_ = 97.8 and *R*_2_ = 98.4%, respectively. The deviation from the material balance during batch membrane filtration of 40% ethanol spent lavender extract was calculated by Equation (5) and found to be 0.703%.

Table 1 summarizes the determined rejection coefficients of the membrane with respect to key biologically active components. The deviations from the material balance calculated by Equation (9) are also reported. The HPLC results confirmed the presence of rosmarinic acid, caffeic acid, and luteolin in the 40% ethanol spent lavender extract. Their concentrations in the feed, retentate, and permeate are presented in Table 1.

All arithmetic mean values for the membrane rejection coefficients were greater than 93%, confirming the sufficient selectivity of the X201 reverse osmosis membrane against all target compounds in the liquid extract. Among the three analyzed biologically active constituents, rosmarinic acid was present in significantly higher concentration in the liquid extracts, which made it a good choice for a modeling compound. Moreover, the deviation from the material balance of only 0.496% with respect to rosmarinic acid validates the *R*_5_ value of 98.9%, obtained from Equation (8). The membrane performance with respect to the modeling compound can be extrapolated over all remaining related substances and streamline the process design and scale-up, especially when using process simulation environments [43]. Indeed, the R_3_ and R_5_ values for rosmarinic acid well correspond to the *R*_1_ and *R*_2_ values determined for the global antioxidant activity of the extract. At this high rejection rate with a degree of feed volume reduction defined as *V_F_/V_R_* [44] equal to 2, expectedly, the retentate concentrations of all three target compounds were up to two times higher than those in the spent lavender extract, reaching values of 678, 39.3, and 6.05 mg/L for rosmarinic acid, caffeic acid, and luteolin, respectively.

The membrane rejection coefficient according to Equation (7) serves as an approximation of the actual observed rejection coefficient under the assumption of steady-state conditions, as it was calculated from the instantaneous ratio of concentrations in the permeate and retentate. Its values for the rosmarinic acid are in agreement with the *R*_5_ values, which once again validates the accuracy of the experimental determination of the membrane rejection coefficients.

The results obtained for *R*_5_ Equation (8), which was derived based on the material balance of the unit operation) of caffeic acid and luteolin, are considered unreliable. Equation (8) is not applicable for calculating the rejection of caffeic acid and luteolin, due to the deviation from the material balance with respect to these components as well as the low concentrations of luteolin resulting in high uncertainty in its quantitative determination. Therefore, conclusions on the membrane selectivity with respect to caffeic acid and luteolin were drawn only based on the *R*_3_ and *R*_4_ values.

The results obtained from both methods for determining the membrane rejection coefficients demonstrate sufficiently high values for the separation capability of the X201 membrane. This supports its application for concentrating antioxidants in extracts derived from spent lavender.

The GC analysis of the ethanol content in the solvent for extraction and of the permeate and retentate fractions from its membrane filtration confirmed their identical composition of 40 vol% ethanol, which confirmed that the membrane exhibited no selectivity toward the solvent components.

### 3.2. Permeate Flux Determination

**Figure 3:**
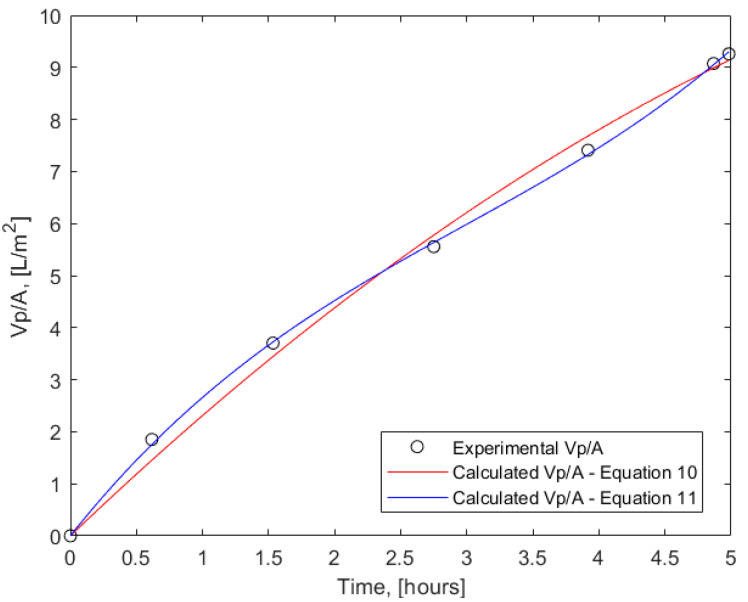
Cumulative permeate volume per unit membrane surface area versus collection time; points—experimental data, curves—calculation via Equations (10) and (11).

Experimental data on the cumulative permeate volume over the collection time are shown in Figure 3. If modeled by a second-order polynomial (Equation (10)), the data revealed a consistent trend of decreasing permeate flux over time, while using a third-order polynomial (Equation (11)) suggested a sigmoidal shape of the correlation.

(10) $$\frac{V_{p}}{A}=at+bt^{2}$$

(11) $$\frac{V_{p}}{A}=at+bt^{2}+ct^{3}$$

A nonlinear regression in the environment of MATLAB R2024a, using Equation (10) as custom equations resulted in a correlation with values for the coefficients a = 2.4298, b = −0.1197, and a sufficiently high coefficient of nonlinear regression of 0.9952. A slightly better description of the experimental points with a coefficient of nonlinear regression of 0.9997 and coefficients a = 3.1907, b = −0.5991, and c = 0.0669 is illustrated when using Equation (11). However, such an unusual behaviour, suggesting the presence of an inflection point where the trend for the permeate flux evolution with time changes from decreasing to increasing during the batch membrane concentration process, cannot be readily explained. The experimental data reported here are not sufficient to prove or disprove the existence of such a feature, which will be investigated in future studies.

The flux at certain points in time, $J_{p}$, was determined by differentiating Equation (10) to derive Equation (12):

(12) $$J_{p}=\frac{dV_{p}}{A\;dt}=a+2bt$$

**Figure 4:**
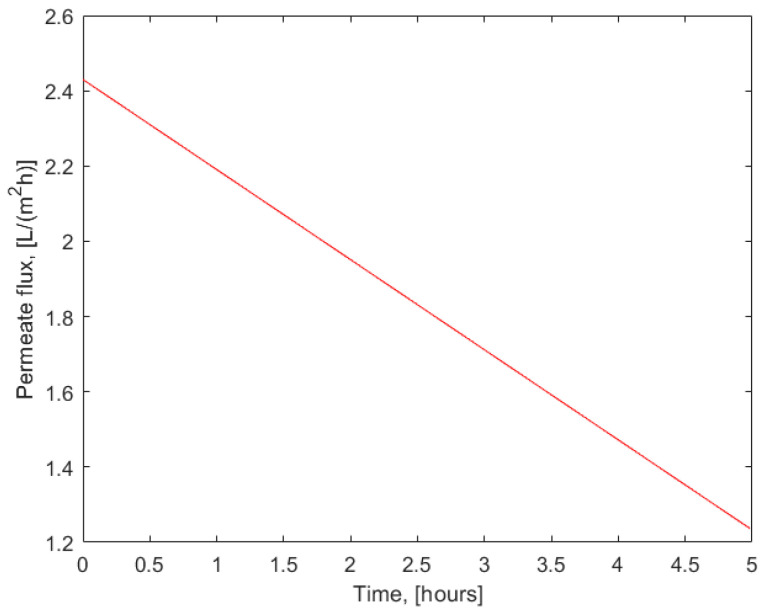
Permeate flux (L/m^2^ h) versus time (hours) during the batch filtration of the extract of spent lavender at 20 bar transmembrane pressure using X201 RO membrane in accordance to Equation (12).

Permeate flux evolution during membrane filtration of the extract from the spent lavender with the X201 reverse osmosis membrane described by Equation (12) is shown in Figure 4.

### 3.3. Process Resistance Due to Osmotic Pressure and Concentration Polarization

The effect of RA extract concentration on process resistance during batch membrane filtration, attributed to osmotic pressure and concentration polarization, was evaluated previously and reported in [42]. Since no membrane fouling or cake formation was observed in the present work, the additional resistances (on top of the intrinsic membrane resistance) due to the osmotic pressure difference across the membrane, *R_O_*, and concentration polarization in the retentate, *R_P_*, as a function of time, were calculated using Equation (13) [42].

(13) $${\left(R_{o}+R_{p}\right)}_{t}=∆P\;\left(\frac{1}{J_{p}}-\frac{1}{J_{s}}\right)$$

The current permeate flux, *J_P_*, was determined using Equation (12), and the solvent flux, J_S_, was measured after completing membrane conditioning with 40% ethanol (*J_S_* = 11.7 L/m^2^ h). In a traditionally reported form of the resistance in a series model [45], the unit of the resistances was m^−1^. In our case, all resistances were multiplied by the viscosity of the solutions and thus the units were kg m^−2^h^−1^. Since membrane separation did not affect the solvent’s ethanol concentration and the extracts were rather dilute hydroalcoholic solutions, the viscosities of the pure solvent, extract, and its fractions were considered to be a constant. Therefore, the modified version of the model, represented by Equation (13), is justified.

Owing to the high rejection rate of the membrane, the permeate concentration was considered to be zero. Thus, the current retentate concentration *C_R_* at any given time was determined using the following equation:

(14) $$C_{R}\left(t\right)=C_{F}\;\frac{V_{F}}{V_{F}-A\;(at+{bt}^{2})}$$

**Figure 5:**
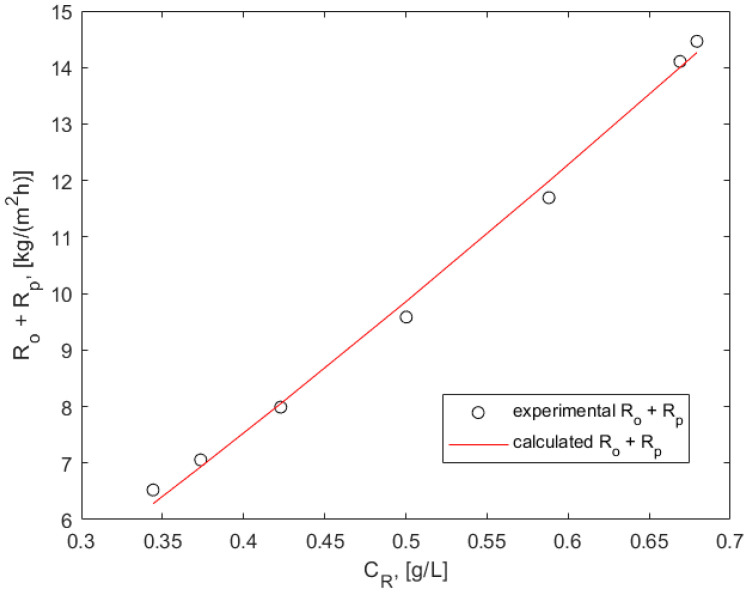
Additional resistance due to osmotic pressure differences and concentration polarization versus the current retentate concentration; points represent the experimental data, while the curve illustrates calculations based on Equations (13) and (14).

The combined process resistance (*R_O_* + *R_P_*) versus the current retentate concentration *C_R_(t)* during the batch membrane filtration was calculated from Equations (13) and (14) and plotted in Figure 5.

The obtained straight line in a double logarithmic scale suggests a relationship of the form

(15) $$R_{O}+R_{P}=k\;C_{R}^{n}$$

in which n represents the slope of the line. The values for the parameters, *k* = 22.7 and *n* = 1.20, were obtained from a nonlinear regression of the experimental data with a coefficient of nonlinear regression of 0.9955. A comparison with previously reported data [38] revealed that the *n* value derived for both a model solution of rosmarinic acid and hydroalcoholic rosmarinic acid-containing extracts from lemon balm was practically equal to that found for the spent lavender extract in this study. This finding supports a hypothesis claimed previously [42], that when the slope, *n*, for a modeling component of an extract is known, it can be used to approximately predict the membrane flux at different concentrations of the key component in the retentate after an initial measurement at a known concentration (to define the intercept, represented by *k*), by drawing the extract line by means of the known slope and estimating the process resistance at any other component concentration.

### 3.4. Antibacterial Activity of Spent Lavender Extract and Its Reverse Osmosis Fractions

**Figure 6:**
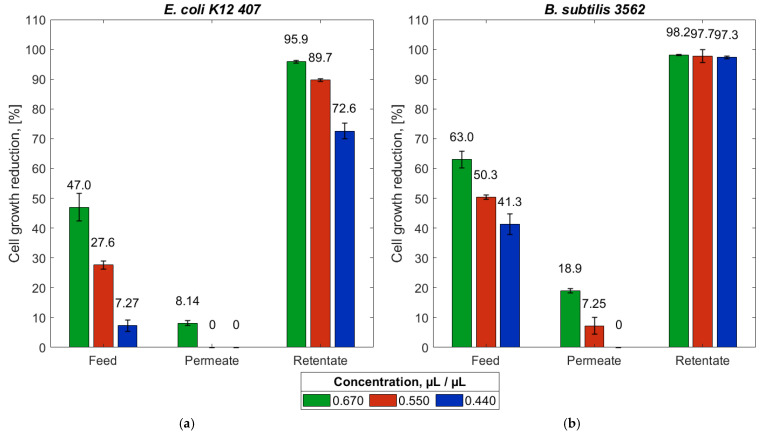
Results of the broth microdilution method for the determination of the reduction in cell growth [%] of *E. coli K12 407* (**a**) and *B. subtilis 3562* (**b**) in spent lavender extract and its fractions obtained after separation with the X201 reverse osmosis membrane.

The antibacterial activity of the spent lavender extract and its fractions (permeate and retentate) were tested against *B. subtilis 3562* and *E. coli K12 407* (Figure 6).

The results of the antibacterial assay indicate that the spent lavender extract was more effective against the Gram-positive bacterium *B. subtilis 3562* than against the Gram-negative bacterium *E. coli K12 407*. The experimental data on growth inhibition demonstrate that the retentate exhibited the highest activity against both tested strains, with inhibition ranging from 73% to 96% for *E. coli K12 407* and from 97% to 98% for *B. subtilis 3562*. A decrease in concentration correlated with a reduction in inhibition across all tested solutions and strains. A clear trend was observed, with increasing activity in the following order: permeate, feed, retentate. The highest antibacterial activity of the retentate and the negligible activity of the permeate against the tested strains confirmed the capability of the X201 reverse osmosis membrane to also retain the antibacterial components of the spent lavender extract at 20 bar. These findings align with the literature reports indicating that rosmarinic acid, caffeic acid, and luteolin exhibit growth-inhibitory effects against *B. subtilis* and *E. coli* strains [46,47,48].

## 4. Conclusions

This study confirmed the applicability of membrane filtration technology for valorizing spent lavender generated during lavender essential oil production.

Batch membrane filtration experiments were conducted with a 40% ethanolic extract of spent lavender as a feed solution, using the commercially available X201 reverse osmosis membrane (TriSep, (Goleta, CA, USA)). The membrane achieved rejection coefficients ranging from 93% to 100% for key biologically active compounds present in the spent lavender extract, including rosmarinic acid, caffeic acid, and luteolin.

The experiments were performed at room temperature and an operating pressure of 20 bar, during which the X201 reverse osmosis membrane demonstrated sufficient permeate flux in the range from 1.24 to 2.43 L/m^2^ h. Importantly, no membrane fouling or cake formation was observed, which is a prerequisite for further research on the process design and scale-up of batch membrane concentration of valuable natural antioxidants contained in 40% ethanol liquid extract from spent lavender.

Furthermore, the process resistance of the membrane filtration due to solute osmotic pressure and concentration polarization followed the pattern of a power-low function with respect to the retentate concentration of rosmarinic acid. The power value obtained in this work for the 40% ethanol extract of spent lavender was practically equal to those values previously reported for a rosmarinic acid model solution and lemon balm hydroalcoholic extracts, indicating the broader applicability of the modeling approach for the prediction of the process resistance and therefore permeate flux at different retentate rosmarinic acid concentrations.

Antibacterial assays demonstrated that the spent lavender extract was more effective against the Gram-positive bacterium *B. subtilis 3562* than against the Gram-negative bacterium *E. coli K12 407*. Across both bacterial strains, the retentate emerged as the most active solution, followed by feed and permeate. This demonstrates that, in addition to retaining the antioxidants in the extract, the membrane is also capable of retaining its antibacterial constituents.

## Figures and Tables

**Table 1:** Experimental data for membrane filtration of 40% ethanol extract from spent lavender using X201 reverse osmosis membrane, at an operating pressure of 20 bar.

Component	Fraction	Concentration, mg/L	Membrane Rejection, %			Deviation from Material Balance, %
			R_3_, %	R_4_, %	R_5_, %	
Rosmarinic acid	Feed	344	98.1	99.5	98.9	0.496
	Permeate	6.59				
	Permeate E	3.72				
	Retentate	678				
Caffeic acid	Feed	19.0	92.6	96.2	106	7.11
	Permeate	1.40				
	Permeate E	1.50				
	Retentate	39.3				
Luteolin	Feed	2.71	100	100	117	11.6
	Permeate	0				
	Permeate E	0				
	Retentate	6.05				

## Data Availability

The data presented in this study are available on request from the corresponding author.
